# Radical antegrade modular pancreatosplenectomy (RAMPS) versus standard retrograde pancreatosplenectomy (SRPS) for resectable body and tail pancreatic adenocarcinoma: protocol of a multicenter, prospective, randomized phase III control trial (CSPAC-3)

**DOI:** 10.1186/s13063-023-07456-0

**Published:** 2023-08-17

**Authors:** Jialin Li, Si Shi, Jiang Liu, Chen Liang, Jie Hua, Qingcai Meng, Hang Xu, Miaoyan Wei, Bo Zhang, Jin Xu, Wei Wang, Xianjun Yu

**Affiliations:** 1 Department of Pancreatic Surgery, Fudan University Shanghai Cancer Center, Shanghai, 200032 China; 2 Department of Oncology, Shanghai Medical College, Fudan University, Shanghai, 200032 China

**Keywords:** Distal pancreatectomy, Clinical trials, Pancreatic cancer, Pancreatic surgery, Radical antegrade modular pancreatosplenectomy

## Abstract

**Background:**

Pancreatic ductal adenocarcinoma (PDAC) is a highly aggressive malignancy. Radical surgical resection offers the only potential cure. There is increasing agreement that radical antegrade modular pancreatosplenectomy (RAMPS) may benefit patients with tumors in the body and tail of the pancreas. To address this, the Chinese Study Group for Pancreatic Cancer (CSPAC)-3 trial is proposed to compare the effect of RAMPS and standard retrograde pancreatosplenectomy (SRPS) on patient survival and preoperative safety

**Methods:**

The randomized controlled trial will be multicenter and two-armed with blinded outcomes and intention-to-treat analysis. Three hundred patients with resectable body and tail pancreatic adenocarcinoma will be enrolled and randomly assigned to RAMPS or SRPS. Adjuvant chemotherapy based on an initial regimen will be recommended 4–6 weeks after surgery if no serious complication occurs. The hypothesis that RAMPS improves survival outcomes compared with SRPS will be tested using a superiority trial. The primary outcome will be overall survival (OS). Secondary outcomes will include recurrence-free survival (RFS), R0 resection rate, the number of harvested lymph nodes, postoperative complications, and quality of life scores.

**Discussion:**

The use of RAMPS has increased over the past decade. It is reported that RAMPS is superior to SRPS in improving both the rate of R0 resection and lymph node yield. Despite these advantages, however, there is little high-level documentation of the superiority of RAMPS in terms of survival and this needs to be investigated. To address this issue, CSPAC has instigated the first prospective, randomized phase III control trials, aiming to explore the optimal surgical strategy for improving the prognosis and OS of patients with left-sided pancreatic cancer

Trial registration

Chinese Clinical Trial Registry ChiCTR2100053844; pre-results. Registered on December 1, 2021.

## Administrative information

Note: the numbers in curly brackets in this protocol refer to SPIRIT checklist item numbers. The order of the items has been modified to group similar items (see http://www.equator-network.org/reporting-guidelines/spirit-2013-statement-defining-standard-protocol-items-for-clinical-trials/).

**Table:** 

Title [1]	Radical antegrade modular pancreatosplenectomy (RAMPS) versus standard retrograde pancreatosplenectomy (SRPS) for resectable pancreatic adenocarcinoma of body and tail: protocol of a multicenter, prospective, randomized phase III control trial (CSPAC-3)
Trial registration {2a and 2b}.	Name of the registry Chines Clinical Trial Registry; Trial registration number ChiCTR2100053844; Date of registration December 1, 2021
Protocol version {3}	Version 2.0; 28st September 2022.
Funding {4}	This research received no specific grant from any funding agency in the public, commercial, or not-for-profit sectors.
Author details {5a}	Jialin Li, Department of Pancreatic Surgery, Fudan University Shanghai Cancer Center, Shanghai 200032, China. E-mail: lijialin@fudanpci.org Si Shi, Department of Pancreatic Surgery, Fudan University Shanghai Cancer Center, Shanghai 200032, China. E-mail: shisi@fudanpci.org Jiang Liu, Department of Pancreatic Surgery, Fudan University Shanghai Cancer Center, Shanghai 200032, China. E-mail: liujiang@fudanpci.org Chen Liang, Department of Pancreatic Surgery, Fudan University Shanghai Cancer Center, Shanghai 200032, China. E-mail: liangchen@fudanpci.org Jie Hua, Department of Pancreatic Surgery, Fudan University Shanghai Cancer Center, Shanghai 200032, China. E-mail: huajie@fudanpci.org Qingcai Meng, Department of Pancreatic Surgery, Fudan University Shanghai Cancer Center, Shanghai 200032, China. E-mail: mengqingcai@fudanpci.org Hang Xu, Department of Pancreatic Surgery, Fudan University Shanghai Cancer Center, Shanghai 200032, China. E-mail: xuhang@fudanpci.org Miaoyan Wei, Department of Pancreatic Surgery, Fudan University Shanghai Cancer Center, Shanghai 200032, China. E-mail: weimiaoyan@fudanpci.org Bo Zhang, Department of Pancreatic Surgery, Fudan University Shanghai Cancer Center, Shanghai 200032, China. E-mail: zhangbo@fudanpci.org Jin Xu, Department of Pancreatic Surgery, Fudan University Shanghai Cancer Center, Shanghai 200032, China. E-mail: xujin@fudanpci.org Wei Wang, Department of Pancreatic Surgery, Fudan University Shanghai Cancer Center, Shanghai 200032, China. E-mail: wangwei@fudanpci.org Xianjun Yu, Department of Pancreatic Surgery, Fudan University Shanghai Cancer Center, Shanghai 200032, China. E-mail: yuxianjun@fudanpci.org LJL and SS contributed equally to this study protocol.
Name and contact information for the trial sponsor {5b}	Xianjun Yu, PhD. Department of Pancreatic Surgery, Fudan University Shanghai Cancer Center; Shanghai Pancreatic Cancer Institute; Pancreatic Cancer Institute, Fudan University. 270 Dong’An Road, Shanghai 200032, China; E-mail: yuxianjun@fudanpci.org.
Role of sponsor {5c}	SS, WW and XJY conceived of and planned the trial. JLL, JL and SS drafted the trial protocol and edited its final version. This trial is under the support of the Chinese Study Group for Pancreatic Cancer (CSPAC). All authors read and approved the final version of the protocol and of the manuscript.

## Introduction

### Background and rationale {6a}

#### Pancreatic cancer

Pancreatic cancer is one of the most lethal and aggressive cancers in the world with a 5-year survival rate of merely 10% [1]. Body and tail adenocarcinoma is associated with poor prognosis, which is often found in the advanced stage because of a lack of specific clinical signs and symptoms in the early stage. Radical surgical resection is the only opportunity to cure this malignant disease. Conventional distal pancreatomy and splenectomy is the standard surgical approach for pancreatic ductal adenocarcinoma in the body or tail. However, the long-term survival of these patients remains unsatisfactory, with published 5-year overall survival rates ranging between 5 and 30% [2–4].

#### Surgery for resectable pancreatic adenocarcinoma of body and tail

SRPS involves dissection of the pancreas and splenic artery at the proximal margin of the tumor, followed by resection of the pancreatic body collar and spleen and clearance of the retroperitoneal tissue. This surgical approach is currently considered to have two limitations: (1) low R0 resection rate at the posterior margin, routine separation of the posterior margin of the pancreas to the dorsal side of the tumor, and lack of sufficient visualization during separation to ensure negative margins; (2) incomplete lymph node dissection, especially lack of lymph node dissection targeting the superior mesenteric artery and peri-abdominal trunk [5, 6].

In 2003, Strasberg et al., from Washington University, described a new distal pancreatectomy technique, termed RAMPS, to increase the rate of R0 resection and lymph node yield for pancreatic cancer in the body or tail. The procedure is performed as follows: the neck of the pancreas and splenic vessels are divided, followed by lymph node and perineural plexus dissection from the celiac axis downward to the SMA. Then, the dissection is continued laterally anterior (anterior RAMPS) or posterior (posterior RAMPS) to the left adrenal gland. Strasberg et al reported that RAMPS could achieve an R0 resection rate of 91% and an overall survival rate of 26% at 5 years after surgery [6].

#### Rationale for the present study

In the past decade, the RAMPS procedure has been increasingly applied. Previous studies have shown that RAMPS significantly improves R0 resection rate and lymph node clearance compared to SRPS. However, reports in recent years have shown that despite the theoretic advantages of RAMPS over SRPS, there is currently no high-level evidence of a survival benefit with RAMPS [7–9]. The potential advantage of RAMPS in terms of survival still needs to be proven.

Based on this status, CSPAC initiated the first prospective, randomized phase III control trials in this field, aiming to analyze the role of surgery of RAMPS in resectable pancreatic adenocarcinoma of body and tail. The hypothesis of this trial is that surgery of RAMPS improves survival in patients with resectable pancreatic adenocarcinoma of body and tail. Both surgery types (RAMPS and SRPS) are proposed to patients in daily practice in different centers in China and therefore randomization is not believed to be a problem. In contrast to phase III trials in which patients are randomized in a placebo or no treatment arm, both randomization arms in this study contain an accepted treatment modality, which can be easily explained to patients. Because patients with locally advanced pancreatic adenocarcinoma of body and tail require different preoperative treatment regimen such as (chemo) radiation therapy and encounter more serious postoperative complications, these patients are excluded from the study.

### Objectives {7}

#### Primary objective

• To compare the overall survival (the time from randomization to death due to any cause or censor) of RAMPS versus SRPS for patients with resectable body and tail pancreatic ductal adenocarcinoma.

#### Secondary objectives

• To compare the recurrence-free survival (the time from randomization to recurrence or censor) of RAMPS versus SRPS for patients with resectable body and tail pancreatic ductal adenocarcinoma.
• To compare the R0 resection rate of RAMPS versus SRPS for patients with resectable body and tail pancreatic ductal adenocarcinoma.
• To compare the number of harvested lymph nodes of RAMPS versus SRPS for patients with resectable body and tail pancreatic ductal adenocarcinoma.
• To compare the postoperative complications of RAMPS versus SRPS for patients with resectable body and tail pancreatic ductal adenocarcinoma.
• To compare the quality of life (QoL) scores of RAMPS versus SRPS for patients with resectable body and tail pancreatic ductal adenocarcinoma.

### Trial design {8}

In this prospective, multicenter, randomized control study, three hundred patients with resectable pancreatic adenocarcinoma of body and tail will be enrolled and randomly 1:1 assigned to either the RAMPS arm or SRPS arm to investigate the efficacy and safety of the surgery.

## Methods: participants, interventions, and outcomes

### Study setting {9}

**Fig. 1:**
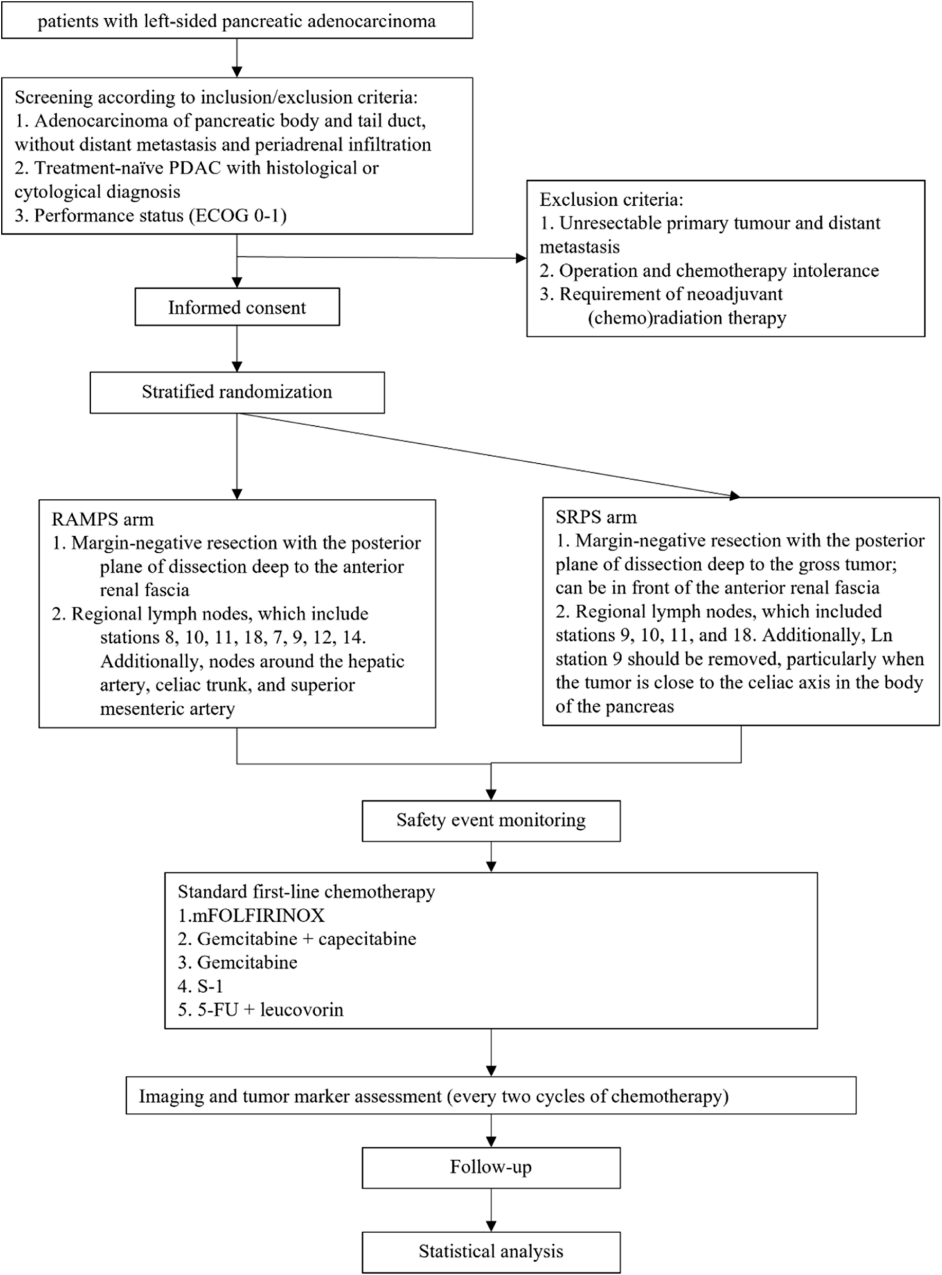
Flow chart

The randomized controlled trial will be multicenter, two-armed, and blinded (both patient- and observer-blinded) to assess the efficacy of RAMPS versus SRPS for the treatment of resectable pancreatic adenocarcinoma of the pancreatic body and tail. A superiority trial will be used to test the hypothesis that RAMPS is superior to SRPS in improving survival outcomes. The full protocol was prepared in line with the Standard Protocol Items: Recommendations for Interventional Trials (SPIRIT) recommendations. Figure 1 shows the flow chart of the study. The complete protocol is attached in Supplementary file.

The trial will be conducted in CSPAC-associated hepatobiliary-pancreatic units or pancreatic cancer centers with high volumes of suitable cases. It will be a multicenter trial. Suitable sites will be selected based on their case volumes, surgical expertise, and surgical oncological experience according to the standards of the protocol. In terms of case volumes, the surgical cases treated should comprise at least 50 and be mostly distal pancreatectomies. A multi-disciplinary team will evaluate the patient regimens.

### Eligibility criteria {10}

#### Inclusion criteria

• Treatment-naïve PDAC diagnosed either histologically or cytologically.
• Adenocarcinoma of the pancreatic body and tail duct, together with an absence of distant metastases (e.g., liver, peritoneum, lung) and periadrenal infiltration as shown by radiological assessment (enhanced computed tomography [CT], enhanced magnetic resonance imaging [MRI], or positron emission tomography-computed tomography [PET-CT]) and surgery.
• The presence of a standard resectable ductal adenocarcinoma in the body and tail of the pancreas, evaluated both preoperatively and intraoperatively (refer to NCCN guideline 2021 of Pancreatic Cancer and ISGPS consensus 2014 of standard distal pancreatectomy)
• No indications for neoadjuvant therapy (chemotherapy or radiation).
• No previous systemic therapy for advanced disease.
• No contra-indications for curative surgery.
• Eastern Cooperative Oncology Group (ECOG) performance status of 0 or 1.
• Aged between 19 and 80 years.
• Laboratory findings four weeks before randomization: Adequate bone marrow function (Hb ≥6.0 mmol/L, absolute neutrophil count ≥1.5 × 109/L, platelet count ≥100 × 109/L), renal function (serum creatinine ≤ 1.5 × ULN and creatinine clearance, Cockroft formula, ≥30ml/min), liver function (serum bilirubin ≤ 2 × ULN, serum transaminases ≤ 3 × ULN).
• Expectation of adequate follow-up
• Written informed consent

#### Exclusion criteria

• Pregnancy, lactation.
• Primary tumor deemed unresectable due to, e.g., neurovascular encasement, significant ingrowth in the pancreatic head.
• Conditions precluding safe or feasible resection of the primary tumor, e.g., massive ascites.
• Intraoperative exclusion criteria including evidence of metastasis, non-pancreatic primary disease, unresectable tumor, and Gerota fascia invasion.
• Postoperative exclusion criterion of lack of pathological confirmation of pancreatic ductal adenocarcinoma.
• History of a different primary tumor 5 years before randomization, excluding basal cell carcinoma of the skin or an adequately treated in situ carcinoma in any organ.
• Any medical condition precluding the safe administration of systemic treatment.
• The presence of cardiopulmonary dysfunction affecting tolerance of surgery.
• Requirements for neoadjuvant therapy (chemotherapy or radiation).
• No informed consent, either due to refusal or withdrawal of consent

### Who will take informed consent? {26a}

The study coordinator will obtain consent from the eligible patients, which will ask for permission for the research team to share relevant data and notice that there is neither anticipated harm nor compensation for trial participation

### Additional consent provisions for collection and use of participant data and biological specimens {26b}

The consent form will request participants’ agreement regarding the utilization of their data in the event of their withdrawal from the trial. Additionally, participants will be asked to grant permission for the research team to share pertinent data with individuals from participating universities or regulatory authorities, as applicable. It is important to note that this trial does not entail the collection and storage of biological specimens.

## Interventions

### Explanation for the choice of comparators {6b}

The use of RAMPS has increased over the past decade. It is reported that RAMPS is superior to SRPS in improving both the rate of R0 resection and lymph node yield. Despite these advantages, however, there is little high-level documentation of the superiority of RAMPS in terms of survival and this needs to be investigated. To address this issue, CSPAC has instigated the first prospective, randomized phase III control trials, aiming to explore the optimal surgical strategy for improving the prognosis and OS of patients with left-sided pancreatic cancer.

### Intervention description {11a}

#### Surgery

**Table:** 

Key element	SRPS	RAMPS
Aim of retroperitoneal dissection	Margin-negative resection with the posterior plane of dissection deep to the gross tumor; can be in front of the anterior renal fascia	Margin-negative resection with the posterior plane of dissection deep to the anterior renal fascia
Extent of lymphadenectomy	Regional lymph nodes, which included stations 9, 10, 11, and 18. Additionally, Ln station 9 should be removed, particularly when the tumor is close to the celiac axis in the body of the pancreas.	Regional lymph nodes, which include stations 8, 10, 11, 18, 7, 9, 12, and 14. Additionally, nodes around the hepatic artery, celiac trunk, and superior mesenteric artery

#### Examination after surgery

The following laboratory examination should be performed after treatment (may overlap the examination prior to the next treatment)

• Blood routine, urine routine, stool routine+occult blood tests
• Liver function (AST, ALT, γ-GT, TBil, albumin, pre-albumin), renal function (creatinine), electrolytes, blood glucose, blood amylase
• Coagulation function (PT)
• Quantitative measurement of CA19-9, CA125, CEA and other tumor markers

#### Chemotherapy regimen

The adjuvant chemotherapy regimen is to the discretion of the local investigator, who may choose from the following schedules:

- Modified FOLFIRINOX: Every 2 weeks: 2-h infusion LV 400 mg/m^2^ followed by an FU 46-h infusion of 2400 mg/m^2^ every 2 weeks, with irinotecan 150 mg/m^2^ as a 2-h infusion and oxaliplatin 130 mg/m^2^ as a 2-h infusion on day 1, administer up to 24 weeks.
- Gemcitabine + capecitabine: Every 4 weeks: capecitabine 1660 mg/m^2^ orally b.i.d. on day 1-21, with GEM 1000 mg/m^2^ intravenously on days 1, 8, and 15, 6 cycles, 6 cycles.
- Gemcitabine: Every 4 weeks: GEM 1000 mg/m^2^ intravenously on days 1, 8, and 15, 6 cycles.
- S-1: Every 6 weeks: S-1 80–120 mg/d orally b.i.d. on days 1–28, administered up to 6 months.
- 5-FU + leucovorin: Every 2 weeks: 2-h infusion of LV (200mg/m^2^/day) followed by a 5FU bolus (400 mg/m^2^/day) and 22-h infusion (600 mg/m^2^/day) for 2 consecutive days every 2 weeks, administered up to 6 months.

### Criteria for discontinuing or modifying allocated interventions {11b}

Subjects may early discontinue treatment or withdraw from the study before the end of the study. Possible causes of the discontinuation/withdrawal may be as below:

• Clinical symptoms and signs, laboratory test results in consistent with the appearance of pregnancy
• Subjects receive unallowed concomitant treatment which may significantly affect the assessment of the effect.
• Intercurrent diseases or other events that may significantly affect the clinical condition and endpoint.
• Poor compliance which may interfere with the analysis of the effect.
• Announcement of withdrawal by the subject or their legal representative.
• Loss to follow up
• Death
• Decision made by the organizer of the study

The follow-up will be continued if a treatment discontinuation is judged by the investigator as an event different from withdrawal, the data of the subject will still be included in the study analysis.

### Strategies to improve adherence to interventions {11c}

After surgery, telephone visit will be performed once 1 month for every subject till the end of the study.

### Relevant concomitant care permitted or prohibited during the trial {11d}

Any operation if deviates the study plan should not be performed without permission. The study organizer should be informed as quickly as possible of any accidental or deliberate deviation (e.g., inclusion/exclusion criteria, dosage, treatment cycle omission)

### Provisions for post-trial care {30}

Other unforeseen or even serious adverse effects may occur with any treatment or medication. The doctor will closely monitor your condition, and if you have an adverse reaction, you must notify your doctor promptly, and your doctor will determine if you have an adverse reaction and will use other drugs to treat you to reduce the adverse reaction or discomfort. In the unlikely event that you develop an unexpected adverse reaction, we will manage it in accordance with clinical study regulations, excluding common expected adverse reactions or chemotherapy-related comorbidities, and medical issues not related to this clinical study.

### Outcomes {12}

#### Primary outcome

The primary outcome is OS (interval between randomization and all-cause death) in all randomly assigned patients.

#### Secondary outcomes

Secondary outcomes included Quality of Life (QoL), RFS, the number of dissected lymph nodes, R0 resection rate, and postoperative complications.

QoL is assessed among patients by using the European Organization for Research and

#### Treatment of Cancer Quality of Life Questionnaire C-30 (EORTC QLQ-C30)

Patients will be invited to finish the two questionnaires at the day of recruitment, the day of charge, and 1^th^, 12^th^, 24^th^, and 36^th^ months after randomization.

RFS is defined as the time from the date of randomization to the day of tumor recurrence, tumor progression, or patients’ death assessed up to 36 months.

## Participant timeline {13}

**Table:** 

Parameter	Pre-study screening	Randomization				Follow-up
		Baseline screening	RAMPS arm	SRPS arm	Examination after surgery	
Written informed consent	✔					
ECOG scoring	✔	✔	✔	✔	✔	✔
Abdominal CT/MRI scanning (plain+enhanced)	✔	✔				✔
Chest CT plain scanning	✔	✔				✔
Quantitative measurement of CA19-9, CA125, CEA	✔	✔	✔	✔	✔	✔
Demographic data	✔					
History and physical examination	✔	✔				
Blood routine, biochemical and coagulation function tests	✔	✔	✔	✔	✔	✔
Blood, urine, stool samples for lab test		✔				
EKG		✔				
QoL scoring		✔	✔	✔	✔	✔
Concomitant treatment			✔	✔		
Adverse events		✔	✔	✔	✔	✔
Inclusion criteria		✔				
Randomization		✔				
Urine pregnancy test		✔				
Operation method			✔	✔		
Adjuvant chemotherapy and dosage			✔	✔		
Recurrence/metastasis/progress of tumor					✔	✔

### Sample size {14}

Based on the former small sample study results (the expected median survivals were 24.6 months and 15.5 months in the operation group and control arm, respectively), and the assumption that the enrollment requires 24 months, the last patient should be followed at least 12 months, a total of 266 patients (133 vs 133) is needed according to PASS 15.0 software (two-sided Log-Rank test, significance 0.05; power 80%). Considering 10% drop-off, 296 patients is needed. Therefore, we plan to enroll 300 patients (150 each group) that will be sufficient to meet the needs of the sample size.

### Recruitment {15}

In principle, all eligible patients (according to inclusion/exclusion criteria) will be enrolled. The enrollment and its completion in each surgery group will be summarized with a list of drop-off. The comparison of different data set size, case distribution, total drop-off rate, and cause of termination in each group will be listed in detail. The demography characteristics (age, height, vital signs, etc.), history, as well as medication history will be described, and a comparison of age, height, and weight, etc., between the groups will be performed to assess the comparability of the two groups

### Assignment of interventions: allocation

#### Sequence generation {16a}

Patients will be enrolled based on inclusion/exclusion criteria, baseline examination, imaging, and pathological examination (reviewed by a third party). The general information (initial, age, gender, screening number), which is obtained via the IVRS/IWRS system, and the stratification variable of the subjects will be used to get a random number. Eligible individuals were randomized to the RAMPS group or SRPS group by the coordinating center using a computer-generated randomization list (permuted blocks with randomized block sizes 2, 4, 6, or 8 to ensure equal numbers in both groups). The date when the random number is generated will be defined as day 0 to count the study date. A copy of the ID card will be filed for every enrolled patient.

### Concealment mechanism {16b}

Allocation concealment will be ensured using sequentially numbered opaque envelopes prepared previously by a person independent from recruitment or allocation of participants to groups.

### Implementation {16c}

The allocation sequence was generated by the study statistician and tested in the online randomization system. The principal investigators and enrolling study staff are unaware of the sequence.

## Assignment of interventions: blinding

### Who will be blinded {17a}

Trial participants, care providers, outcome assessors, and data analysts will be blinded to the assignment of intervention. The only research personnel who will be unblinded are the surgeons performing the RAMPS or SRPS. The surgery records and surgery videos will be strictly kept confidential, they will not be distinguishable to the trial participants, care providers, or outcome assessors to maintain their blinding. Data analysis will occur outside of the unit with the type of intervention received by each patient concealed until the completion of analysis.

### Procedure for unblinding if needed {17b}

If needed, all trial staff (trial participants, care providers, outcome assessors, data analysts, and surgeons) can get access to the subject randomization list and the total dispensing unit number list, and perform the unblinding procedure. Unblinding can be performed under the following circumstances: treatment of a participant in a medical emergency that requires knowledge of treatment allocation; treatment of a participant for an adverse event (AE); in the event of a suspected unexpected serious adverse reaction (SUSAR); for the submission of trial data to the Data Monitoring and Safety Committee for the monitoring of safety and/or efficacy.

## Data collection and management

### Plans for assessment and collection of outcomes {18a}

#### Evaluation of outcomes

## Efficacy variables

• Major parameter: OS
• Minor parameters: RFS, the rate of R0 resection, the number of lymph node dissection, postoperative complications, and QoL scores.

### Procedure

## Primary efficacy analysis

Primary efficacy analysis is to determine the overall survival (OS): log-rank test will be performed to compare the differences between the RAMPS group and the SRPS group. Kaplan-Meier curve will be used to plot the difference in OS.

## Secondary efficacy analysis

Recurrence-free survival (RFS) will be analyzed using the same method as that for OS. Descriptive analysis using the covariance analysis (ANCOVA) model will be performed for the changes in QoL when compared with baseline. Baseline serves as a covariant in the model, and the effects of assessors are considered as a random effect. The value of the difference (D-value) in each group before and after treatment, the LSmeans of the difference in D-values and the 95% confidential interval of the difference between the two groups are calculated based on the model.

The incidence and severity of hematological, non-hematological, and overall toxicity and complications will be assessed. The incidence of any Grade 3 or 4 toxicities will be compared across treatment groups using Pearson’s chi-square test with continuity correction or Fisher’s exact test where appropriate.

For the rate of R0 resection and number of harvested lymph nodes outcome, two-sided tests will be performed for all statistical tests, and *p*-value <0.05 will be considered a statistical difference. Mean value, standard deviation (SD), median, minimum, maximum, upper, and lower quartiles will be calculated for quantitative index description, while a number of cases and percentage will be used to describe the classification index. Comparison of the general condition of the two groups will be performed by the corresponding analytic method according to the type of the index: paired-t-test or Wilcoxon rank-sum test will be used for comparison of quantitative data between the groups, chi-square test or Fisher’s exact probability test will be used for classification index, and Wilcoxon rank-sum test and CMH test for ranked data.

For the QoL outcome, characteristics of the RAMPS group and SRPS group will be compared, and potential biases assessed. Quality of life will be assessed over time and surgery groups compared using longitudinal analysis with appropriate recognition for informative dropout. Joint modeling or quality-adjusted survival analysis will be undertaken to allow a simultaneous assessment of the quality of life and survival. The changes in QoL before and after surgery in each group are compared using paired-sample *t*-test.

Analyses will be done in eligible patients according to the intention-to-treat principle.

## End of trial (EOT) assessment

EOT assessment will be performed for every subject who has completed the study at the end of the trial, including:

- Physical examination, weight, vital signs, ECOG scoring, QoL scoring
- Blood routine, liver, and renal function tests
- Imaging examination (according to the length of follow-up duration)
- CA19-9, CA125, CEA, and other tumor markers
- Assessment of adverse events.

### Plans to promote participant retention and complete follow-up {18b}

A regular telephone follow-up will be performed every 2 months in the participating centers.

### Data management {19}

#### Treatment of the data

The data of enrolled subjects must be recorded by the investigator in the CRF after randomization, data of those who fail to pass the screening is not required to be filled in. The accuracy and completion of the data must be ensured, and the original records must be well kept. The CRF for every enrolled patient must be completed right in time. The completed CRF will be reviewed and then turned over to the data administrators for data entry and management.

#### Data entry

Data entry and management are performed by the appointed data administrator unit. Data administrators compile data entry procedures by computer software to perform data entry and management. Transcribed data must be proofread by another person to ensure the accuracy of the data.

#### Medical information coding

The coding of medical information will be performed by using:

• MedDRA 11.0 (patient history and adverse event)
• WHO Drug 2008.03 (concomitant medications)
• NCI-CTCAE 3.0 (toxic reaction)

#### Data review

The established database will be reviewed by the principal investigator, organizer, data administrators, statisticians, and the locking of the database will be performed when the study data set and the statistical analysis plan have been confirmed.

### Confidentiality {27}

Any observed results and examination results during the study must be completely and accurately recorded in a standard manner in the medical record and CRF by the investigator right in time. At will modification of the records is not allowed. Correction of wrong-filled items must keep the original record legible, and the correction must be dated along with the signature of the modifier’s name. The original data/file must be carefully stored in the research center according to the ICH GCP guidelines as well as local laws and regulations.

The superior competent department and the regulatory authorities have the right to supervise and inspect the implementation process of the clinical study as well as the original data/source file but have no right to modify the original data/source file. Once a mistake is found, the investigator must be informed because s/he is the only one who has the right to modify the record.

### Plans for collection, laboratory evaluation, and storage of biological specimens for genetic or molecular analysis in this trial/future use {33}

#### Standard operating procedure for sample collection

Samples from the subjects are collected strictly according to our center’s standard operating procedure (SOP) for blood and tumor tissue sample collection.

#### Sample storage

The principal investigator will be responsible for the storage of samples. Informed content is obtained prior to the enrollment. Both paraffin-embedded and frozen specimens of tumor tissues will be regularly kept after surgery in the operation group; the paraffin-embedded will be used to make tissue chip after completion of enrollment. The blood samples will be centrifuged to separate serum and blood cells for storage.

#### Statistical methods

### Statistical methods for primary and secondary outcomes {20a}

#### Statistical methods

The study statistical analysis plan is made by a professional statistician with the principal investigator when the study plan is determined. SPSS 25.0 software will be used for statistical analysis. Two-sided tests will be performed for all statistical tests, and *p*-value <0.05 will be considered a statistical difference. Mean value, standard deviation (SD), median, minimum, maximum, and upper and lower quartiles will be calculated for quantitative index description, while a number of cases and percentage will be used to describe the classification index. Comparison of the general condition of the two groups will be performed by the corresponding analytic method according to the type of the index: paired-*t*-test or Wilcoxon rank-sum test will be used for comparison of quantitative data between the groups, chi-square test or Fisher’s exact probability test will be used for classification index, and Wilcoxon rank-sum test and CMH test for ranked data. RFS serves as the first minor efficacy variable, which is tested only when a significant difference in OS (the major efficacy variable) is found.

#### Primary efficacy analysis

Primary efficacy analysis is to determine the overall survival (OS): log-rank test will be performed to compare the differences between the RAMPS group and the SRPS arm. Kaplan-Meier curve will be used to plot the difference in OS.

#### Secondary efficacy analysis

Recurrence-free survival (RFS) will be analyzed using the same method as that for OS. Descriptive analysis using the covariance analysis (ANCOVA) model will be performed for the changes in QoL when compared with baseline. Baseline serves as a covariant in the model, and the effects of assessors are considered as a random effect. The value of the difference (D-value) in each group before and after treatment, the LSmeans of the difference in D-values and the 95% confidential interval of the difference between the two groups are calculated based on the model. The changes in QoL before and after treatment in each group are compared using paired-sample *t*-test.

### Interim analyses {21b}

The trial, CSPAC-3, will be subject to oversight by the Data Monitoring Committee (DMC), which will evaluate trial data in light of global evidence. The DMC has planned three interim analyses, each to be conducted after 75 (25%), 150 (50%), and 225 (75%) patients have been enrolled and followed for at least 90 days. During each interim analysis, the Safety Data Monitoring Committee will review unblinded data pertaining to primary and secondary objectives in both arms of the study. The trial will be stopped or amended if sufficient evidence emerges that one or other treatment is clearly indicated or contra-indicated, as considered by the DMC in light of the presented analyses. Analyses will be reported to DMC members who will consider the data in a clinical context accounting for other emerging worldwide evidence and overall clinical relevance. Subsequently, DMC members shall provide formal recommendations to the trial working group concerning the recruitment of patients into the study, adhering to a trial-specific DMC charter in accordance with ICH GCP guidelines.

### Methods for additional analyses (e.g., subgroup analyses) {20b}

The safety analysis includes all AEs and SAEs (including the number and the occurrence rate of the events), complications and mortality during the perioperative period, routine blood and biochemical data, weight, vital signs, physical examinations, and all treatments and simultaneous medication. Safety indexes include weight, vital signs, clinical laboratory indexes, AE, etc. The laboratory indexes will be listed according to the treatment group, actual value at each assessment, change in value than baseline, and comparison with the reference range (lower than, within, or higher than the reference range); the weight and vital signs will be listed according to the treatment group, actual value at each assessment, and change in value than baseline.

The number of subjects who experiences AE to the number of all patients with assessable safety data is used to represent the occurrence rate of AE. The AE rate in each group will be summarized using the organ classification and standard terminology listed in MedDRA. An AE during the study is defined as an event that occurs during the period from the first dose to the last dose or within 30 days after the last dose or the day when the treatment is discontinued. The severity of the toxic reaction is classified according to NCI- CTCAE 3.0. For an individual subject, the same adverse event if occurs more than once will be counted only once, the event of the worst CTCAE grade is to be counted.

The type, grade, frequency, severity, lasting duration, as well as its relationship to the intervening agent, its treatment and outcome will be listed and described in detail for all AEs.

### Methods in analysis to handle protocol non-adherence and any statistical methods to handle missing data {20c}

In case of missing data, an intention-to-treat analysis will be performed. For primary outcomes, for patients who are lost to follow-up, censored data will be the date of the last follow-up. For secondary outcomes, we may consider multiple imputation or other strategies in place of imputed missing values.

### Plans to give access to the full protocol, participant-level data, and statistical code {31c}

In the current document, the full protocol is outlined. Non-identifiable data may be made available upon reasonable and well-motivated request. A data-sharing agreement must be signed in addition to privacy regulations and informed consent. Data cannot be made freely available to the public.

### Oversight and monitoring

#### Composition of the coordinating center and trial steering committee {5d}

The coordinating center is the Department of Pancreatic Surgery, Fudan University Shanghai Cancer Center, Shanghai, China. The institution possesses extensive expertise in the execution of clinical research. The coordinating center team will furnish participating centers with instruction, direction, and assistance to ensure strict adherence to the research protocol. The team's proficiency and aptitude in research methodologies and requisite biostatistics are well-established. In each participating center, a lead investigator (surgeon) will be identified, to be responsible for identification, recruitment, data collection, and completion of CRFs, along with follow-up of study patients and adherence to study protocol.

The Steering Committee of the study shall assume responsibility for supervising the study, which includes the authority to suspend or modify procedures as deemed necessary, analyze and interpret data, and draft the final manuscript. The Committee is led by a designated chairperson and shall conduct coordination through monthly face-to-face or telephonic meetings.

To ensure optimal quality control regarding GCP-compliant data management, monitoring, and biometry, the Institutional Review Board/Ethics Committee (IRB/EC) is part of the trial. Prior to the initiation of the study, the study plan, informed content, and any potentially wanted or required document should be submitted to the IRB/EC with an enclosed cover or form, listing the names and publication date of the documents submitted, as well as the research center waiting to be permitted. The documents will also be submitted to the regulatory authority according to local laws and regulations.

### Patient and public involvement

There is no patient and public involvement in this study.

### Composition of the data monitoring committee, its role and reporting structure {21a}

A data monitoring committee (DMC) has been established parallel to the finish of the study protocol. The data monitoring committee consists of the key persons for conducting this trial (Jialin Li, Si Shi, and Wei Wang) and will maintain monthly meetings to discuss the progress and possible harms of this trial. The DMC should consider essential parts of the study conduct such as protocol adherence, safety monitoring, and patient withdrawal. In addition, a DMC should consider potential recommendations to the sponsor if major problems with study conduct are observed.

### Adverse event reporting and harms {22}

Adverse event will closely be monitored. Any AE (reported by the subject or observed by health-care providers) from randomization to the end of the study or withdrawal, must be recorded in the medical record and CRF. A record of an AE includes the name of AE, time of occurrence and end, severity, treatment, outcome, relationship with intervening agent, etc.

Changes in vital signs, physical examination results, clinical manifestation, and laboratory tests must be assessed during the study. Any medical record about AE must be recorded in the original document, including laboratory tests and auxiliary examination results. Reported AE and its severity must be assessed according to NCI-CTCAE 3.0.

Relevant researcher’s name and telephone number must be offered to subjects at the time of signing informed content, so that they can contact the researcher in case of an emergency, or report any medical symptom or adverse event.

Toxic reaction or AE exists prior to the study, will be recorded as an AE only when it significantly elevates to a higher level. In this study, the worsening of tumor symptoms than the baseline is also recorded as an AE.

### Frequency and plans for auditing trial conduct {23}

The Ethics Committee will be responsible for monitoring the trial. Audits on accuracy may be carried out at any time and at least twice. The auditing trial conduct was performed by a team independent from the investigators and the sponsor. The trial management group convenes on a monthly basis to assess advancements in recruitment, clinical endpoint monitoring, and data integrity. The Trial Steering Group (TSG) convenes 1 to 2 months following each DMC meeting, which transpires at intervals of approximately 4–6 months. The TSG establishes objectives for recruitment, data aggregation, and adherence to protocol. The TSG conducts a comprehensive review of all grievances related to the trial. The trial statistical analysis plan will be presented to the TSG for endorsement. The TSG considers new information relevant to the trial, including reports from the DMC and the results of other studies that may have a direct bearing on the future conduct of the trial. Annual reports are submitted to the Ethics Committee which document progress in recruitment, SAEs, and protocol deviations and violations.

### Plans for communicating important protocol amendments to relevant parties (e.g., trial participants, ethical committees) {25}

The revision of the study plan is allowed during the study if necessary. The revision must be made by the investigator and the organizer according to relevant GCP requirements. The approval of the Ethics Committee must be obtained before the implementation of the revised plan. The revision opinion approved by the EC must be included in the revised plan.

### Dissemination plans {31a}

The results of the study may be published in medical journals or used in teaching. In addition, the study and its results can be registered in the health research institutes and published at the website of the health care registration authority (e.g., ClinicalTrials.gov), according to the requirements of local health care authority. The selection of the first author is based on several considerations, including but not limited to below items: participate in the study, has contribution to the development of the study plan, and contribute to the manuscript, abstract, description, and analysis.

## Discussion

Traditional distal pancreatectomy and splenectomy for ductal adenocarcinoma in the pancreatic body and tail were standardized by Mayo in 1913 [10]. However, this procedure results in relatively poor lymph node yields, high rates of positive margins, and poor OS [2, 4]. The RAMPS procedure was proposed by Strasberg et al. in 2003 as a modification of the traditional procedure. In contrast to the usual retrograde resection, RAMPS uses a posterior plane of dissection deep to the anterior renal fascia with dissection of the regional lymph nodes together with those of the hepatic artery, celiac trunk, and superior mesenteric artery. This facilitates R0 resection and increased lymph node retrieval.

However, there are no guidelines for the use of RAMPS for adenocarcinoma of the pancreatic body and tail. Lymph-node involvement and R0 resection are reported to be independent risk factors in pancreatic cancer. It is agreed that RAMPS allows the dissection of a greater number of lymph nodes [8, 9, 11–13]. Strasberg et al. proposed the use of RAMPS for N1 lymph node dissection [5]. A previous study has confirmed that RAMPS results in greater numbers of retrieved lymph nodes in comparison with SRPS (28.4 vs 20.7, *P* = 0.0016) [9]. Similar results were reported by Trottman et al with means of 4.3 and 11.2 lymph nodes were dissected in SRPS and RAMPS, respectively (*P* = 0.03) [13]. In terms of R0 resection, Quanyu Zhou et al demonstrated statistically significant R0 resection rates between RAMPS and SRPS (RR = 2.37, 95% CI [1.19 ~ 4.72], *P* = 0.01), finding that while the one-year OS was higher with RAMPS compared with SRPS, tumor recurrence did not differ significantly between the two procedures [7]. Feng Cao et al. conducted a retrospective study on 378 patients and drew a similar conclusion [14]. Although RAMPS is oncologically superior to SRPS for the treatment of left-sided pancreatic cancer, high-grade studies are required to verify the survival benefits of the procedure.

The majority of studies on RAMPS have been retrospective with some discrepancies in the criteria for participant selection. There are also discrepancies in the analysis of resection specimens. Further inconsistencies include the definition of R0, defined in the USA as a 0-mm distance from the tumor margin and a 1-mm distance in many centers in Europe and Australia (excluding datasets prior to 2006) [15, 16]. There has also been poor standardization of the pathological analysis of PDAC specimens before 2008 when Esposito et al. proposed the use of a standard axial slicing procedure with multicolor staining of margins and extended sampling [17].

In addition, the numbers of cases assessed in these studies were small, and geographical differences in preoperative assessments, surgical techniques, and postoperative management add further complications. The use of older datasets also does not allow consideration of recent progress in surgical techniques, perioperative management, and adjuvant therapy. Thus, the indications and survival outcomes are not clear, and further evidence is required to assess whether RAMPS is applicable to all adenocarcinomas of the pancreatic body and tail.

## Trial status

The trial was registered on December 1, 2021. Recruitment of participants started in December 2022, and it is anticipated to be completed by September 2025. The protocol version number and date were 3.0 and September 12, 2022, respectively.

## Data Availability

The de-identified datasets used and/or analyzed during the current study are available from the corresponding author on reasonable request.

## Abbreviations

CRF: Case report form
CSPAC: Chinese Study Group for Pancreatic Cancer
DLT: Dose-limiting toxicity
DMC: Data Monitoring Committee
ECOG: Eastern Cooperative Oncology Group
EOT: End of trial
GCP: Good clinical practice
IRB/EC: Institutional Review Board/Ethics Committee
OS: Overall survival
PDAC: Pancreatic ductal adenocarcinoma
QoL: Quality of life
RAMPS: Radical antegrade modular pancreatosplenectomy
RFS: Recurrence-free survival
SOP: Standard operating procedure
SRPS: Standard retrograde pancreatosplenectomy
